# Construction of a Phytic Acid–Silica System in Wood for Highly Efficient Flame Retardancy and Smoke Suppression

**DOI:** 10.3390/ma14154164

**Published:** 2021-07-27

**Authors:** Zhuoran Chen, Shaodi Zhang, Mengyi Ding, Mingzhi Wang, Xing Xu

**Affiliations:** College of Materials Science and Technology, Beijing Forestry University, Beijing 100083, China; chenzhuoranran@163.com (Z.C.); zshaodi384@163.com (S.Z.); dingmengyi@bjfu.edu.cn (M.D.); daxing007@163.com (X.X.)

**Keywords:** wood, flame retardancy, phytic acid, silica, smoke suppression

## Abstract

The intrinsic flammability of wood restricts its application in various fields. In this study, we constructed a phytic acid (PA)–silica hybrid system in wood by a vacuum-pressure impregnation process to improve its flame retardancy and smoke suppression. The system was derived from a simple mixture of PA and silica sol. Fourier transform infrared spectroscopy (FTIR) indicated an incorporation of the PA molecules into the silica network. Thermogravimetric (TG) analysis showed that the system greatly enhanced the char yield of wood from 1.5% to 32.1% (in air) and the thermal degradation rates were decreased. The limiting oxygen index (LOI) of the PA/silica-nanosol-treated wood was 47.3%. Cone calorimetry test (CCT) was conducted, which revealed large reductions in the heat release rate and smoke production rate. The appearance of the second heat release peak was delayed, indicating the enhanced thermal stability of the char residue. The mechanism underlying flame retardancy was analyzed by field-emission scanning electron microscope coupled with energy-dispersive spectroscopy (SEM-EDS), FTIR, and TG-FTIR. The improved flame retardancy and smoke-suppression property of the wood are mainly attributed to the formation of an intact and coherent char residue with crosslinked structures, which can protect against the transfer of heat and mass (flammable gases, smoke) during burning. Moreover, the hybrid system did not significantly alter the mechanical properties of wood, such as compressive strength and hardness. This approach can be extended to fabricate other phosphorus and silicon materials for enhancing the fire safety of wood.

## 1. Introduction

The natural hierarchical structure of wood, coupled with polymer components, facilitate modification and functionalization in easily accessible top-down approaches, resulting in high-performance wood composites with additional properties such as transparency [1,2,3], electromagnetic shielding, [4,5] super-flexibility [6,7], and energy conversion [8,9]. However, the intrinsic flammability of wood restricts the practical application of these developed materials in many fields with high fire-safety requirements [10]. Wooden materials burn easily when they come in contact with a flame or are exposed to a heat flux. The heat released by the combustion of gases and toxic smoke has caused serious fire catastrophes in history and led to irreparable losses of life and property.

Flame-retardant (FR) treatment can effectively enhance the fire safety of wood. Flame retardant chemicals (FRs) may contain halogens (chlorine and bromine), boron, nitrogen, phosphorus, silicon, magnesium, aluminum, ferrum, or their combinations. In the last decades, halogen-based compounds are the most widely used FRs because of their high efficiencies and low dosages, however, many of them can pose a serious threat to biosystems and humans [11,12,13]. Recent studies have been conducted to develop possible alternatives that are consistent with the current green principles. Thorough studies have been conducted on biomolecules, including starches, proteins, chitosans, and struvites because of their accessibility and renewability [14,15,16]. Owing to their chemical composition and structures, these biomolecules exhibit superior FR features when applied to wood. 

Phytic acid (PA), a bio-resourced flame retardant, has drawn research interest. PA can be extracted from plant tissues such as nuts, oil seeds, legumes, and cereals [17]. As a naturally occurring organic acid with high phosphorus content (28 wt% of phosphorus based on molecular weight), PA can act in a condensed phase by favoring dehydration and char formation in the early stages, improving the flame retardancy of wood [18,19]. However, phosphorus FRs are effective only in treatments with high weight percentage gain. These conditions are related to the low thermal stability of P-catalyzed char at high temperatures [20]. Meanwhile, smoke production increases with the addition of a high phosphorus FR content. One facile technique for improving its flame-retardant and smoke-suppressed efficiencies on wood or wood products is the introduction of other FR components, such as hierarchically porous 4A zeolite [21], zinc borate [22], ferric oxide [23], graphene oxide [24], and nanosilica aerogels [25]. All combinations of the aforementioned components are intended to generate more thermally stable, compact, coherent, and crosslinked char residues. 

Silica sol is a colloidal solution characterized by small particle size, low viscosity, non-toxicity, and odorlessness. It is green and environmentally friendly, and can penetrate wood scaffolds and form a stable silica network structure inside via gelation and drying [26]. Xu et al. [27] prepared wood-SiO_2_ composite by in situ polymerization using vacuum-pressure impregnation technology. The TG results showed that the treatment improved the thermal property of the composites. Xiao et al. [28] treated Scots pine with a dispersion of aluminum oxychloride-modified silica. The incorporation of modified silica did not influence the pyrolysis of wood polymers. Miyafuji et al. [29] observed that treatment of wood with silica particles modified with B_2_O_3_ and P_2_O_5_ caused a considerable char yield increase by a simple burning test with propane gas. Liu et al. [30] found that the flame retardancy of poplar wood was significantly improved with the treatment of boric-acid-modified silica sol. Zhu et al. [25] treated wood with ammonium polyphosphate (APP), ammonium monohydric phosphate (DAP), or ammonium phosphate (AP) and nanosilica sol via two-step impregnation. The treatment of the wood improved the flame retardancy of wood and inhibited the release of smoke and toxic gases.

**Figure 1:**
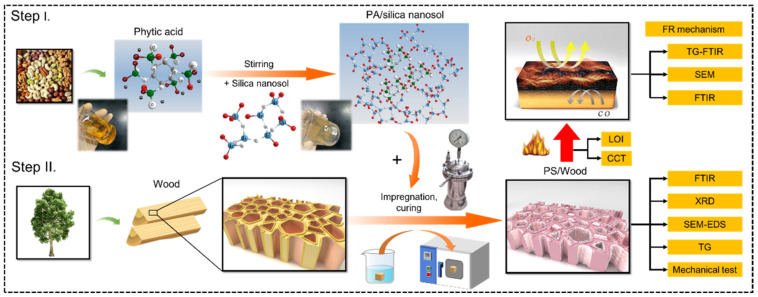
Schematic of an overview on experimental techniques applied in this study.

PA can catalyze the protonation and polymerization processes of Si-O^−^ anions in silica sol because of its strong acidity, and it can bond to silica when temperatures exceed 450 °C [31,32]. Flame-retardant and smoke-suppressed effects of combinations of PA with silica sol on other cellulosic materials, such as wool and cotton, were positive and strong [33,34,35,36]. Fabrics prepared with PA/silica hybrid sol improved in mechanical strength as well [37]. To the best of our knowledge, the flame retardancy and smoke-suppression effects of PA with silica have not been investigated for wood, of which the combustion behavior differs from that of fabrics [38]. In the current study, we constructed a PA–silica hybrid system in wood by a vacuum-pressure impregnation process to improve its flame retardancy and smoke suppression. The system was derived from a simple mixture of PA and silica sol. The flame retardancy and smoke-suppression property, in addition to the mechanical effects of the PA–silica hybrid on wood, were evaluated by LOI, CCT, and mechanical test, respectively, and the underlying FR mechanism was examined by TG-FTIR, SEM, and FTIR here. The schematic of an overview on experimental techniques applied in this study is shown in Figure 1. This study provides a practical basis for the possibility of PA/silica sol as a green flame retardant for wood. The wood prepared with the sol can expand the range of potential applications for renewable and CO_2_-storing wood materials.

## 2. Materials and Methods

### 2.1. Materials

Fast-growing poplar (*Populus cathayana* Rehd) from Liaoning, a province in China, was cut. Sapwood was machined into experimental specimens. The moisture content of the wood specimens was 11.4%. Phytic acid [PA, 70% (*w*/*w*) aqueous solution] was purchased from Macklin Biochemical Technology Co., Ltd. (Shanghai, China); silica nanosol (SiO_2_ content = 30%, pH = 10.46, Na_2_O < 0.1%), which was prepared with sodium silicate via ion exchange technology in industry, was purchased from Peak-tech New Material Co., Ltd. (Shandong, China); and deionized water was purchased from the Institute of Semiconductors (Beijing, China).

### 2.2. Preparation of PA/Silica Nanosol

PA solution was diluted to 20% (*w*/*w*) with water and then added to silica nanosol at a ratio of 1:1 (*w*/*w*). PA catalyzed the protonation and polymerization processes of Si-O^−^ in the silica sol, and it comprised 10% (*w*/*w*) of the mixture. The mixture was then vigorously stirred until it turned to a homogeneous state. 

### 2.3. Wood Impregnation and Curing 

The samples were first oven-dried at 103 °C until constant weight. The dried mass and volume of each were recorded. For comparison, the samples were divided into three experimental groups: Pristine Wood, PA/Wood, and PS/Wood. Pristine Wood (the control group) was impregnated with deionized water; PA/Wood was impregnated with 10% (*w/w*) solution of PA; and PS/Wood was impregnated with the PA–silica nanosol (Table 1). Vacuum-pressure impregnation was adopted to allow the sol/solution to penetrate the wood. Under this method, dried samples were placed in a beaker subjected to a vacuum of −0.09 MPa for 30 min. The sol/solution was injected into the beaker, and 0.5 MPa pressure was exerted for 2 h, followed by a room pressure for 24 h. The samples were then removed from the beaker, and the surface of each sample was wiped with tissue paper. After impregnation, the wood samples were aged under ambient conditions for 2 d, followed by heating at 80 °C for 3 d. The gels were formed in the wood cells, and the wood samples were dried during this process. The constant mass and volume of each sample were recorded. 

### 2.4. Physicochemical Properties of the Impregnated Solution

The pH value of the impregnated sol/solution was detected by a pH meter (PHS-3E, INESA Scientific Instrument Co., Ltd., Shanghai, China). The average size of PA/silica particles in the sol was measured using a laser particle size analyzer (Delsa Nano C, Beckman Coulter, San Diego, CA, USA).

### 2.5. Physical Property Tests 

Wood blocks measuring 20_R_ mm × 20_T_ mm × 20_L_ mm (five replicates per group) were used to calculate the weight percentage gain (WPG) and bulking coefficient (BC). These two physical factors were calculated as follows:

(1) $$WPG\left(\%\right)\;=\frac{M-M_{0}}{M_{0}}\times 100\%$$

where *M* and *M*_0_ are the oven-dried masses of wood specimens after and before the treatment, respectively.

(2) $$BC\left(\%\right)\;=\frac{V-V_{0}}{V_{0}}\times 100\%$$

where *V* and *V*_0_ are the volumes of wood specimens after and before the treatment, respectively.

In addition, the thermal conductivities along tangential section of the wood panels (100_R_ mm × 10_T_ mm × 100_L_ mm, three replicates per group) were measured using a steady-state thermal conductivity meter (Isomet 2114, Applied Precision Co., Ltd., Bratislava, Slovakia) at room temperature. 

### 2.6. Chemical Structure Analysis 

Chemical bonds were studied via Fourier transform infrared (FTIR, Nicolet iS10, Thermo Nicolet Corporation, Madison, WI, USA) spectroscopy in the 4000–400 cm^−1^ range with a resolution of 4 cm^−1^ after 64 scans. The crystalline structure was characterized by X-ray diffraction (XRD, D8 ADVANCE, Bruker AXS, Karlsruhe, Germany) with CuKα radiation at 40 mA and 40 kV. The data were recorded in the 2θ range of 5°–50° with an angular step size of 0.02°. By reference to the Segal method, the relative crystallinities (CrI) of the wood samples were calculated from the following Formula (3) [39]. Prior to the XRD detection, the wood was ground into 100-mesh flour; and prior to the FTIR detection, the wood was finely ground into 200-mesh flour.

(3) $$CrI\left(\%\right)\;=\frac{I_{002}-I_{AM}}{I_{002}}\times 100\%$$

where *I*_002_ is the height of 002 peak (2θ ≈ 22°), and *I_AM_* is the minimum between the 002 and 101 peaks (2θ ≈ 18°). *I*_002_ represents both crystalline and amorphous materials in wood, and *I_AM_* represents amorphous materials only.

### 2.7. Morphological Observation and Element Analysis

The overall appearances of the wood samples were recorded. The micromorphology of the wood and the distribution of the PA and the PA/silica hybrid in the wood structure, were simultaneously observed by field-emission scanning electron microscope (FE-SEM, SU8010, Hitachi, Tokyo, Japan) at an acceleration voltage of 3–5 kV, coupled with energy-dispersive spectroscopy (EDS, MC1000, Hitachi, Tokyo, Japan) at an acceleration voltage of 10 kV. Slices measuring approximately 5_R_ mm × 5_T_ mm × 1_L_ mm were cut off from the middle of the wood samples by using a feather microtome blade (3–70, Feather Safety Razor Co., Ltd., Osaka, Japan). The observed surfaces had been sputtered with a thin gold layer before the test.

### 2.8. Thermal Degradation Analysis 

For a thorough analysis of the thermal degradation of the wood, thermogravimetric (TG) tests were conducted with a TG analyzer (TGA, TG 209F3, NETZSCH, Selb, Germany) in a nitrogen atmosphere and an air atmosphere by using a heating rate of 10 °C/min from 35 °C to 800 °C. The wood was ground into a 200-mesh flour prior to the TG test.

### 2.9. Combustion Property Tests 

In accordance with GB/T 2406-2009, we obtained the limited oxygen indexes (LOI) of the woods (6_R_ mm × 3_T_ mm × 150_L_ mm) with an oxygen index apparatus (M606B, Shanfang Instrument Co., Ltd., Qingdao, China). A cone calorimetry test (CCT) was also performed using a cone calorimeter (FTT00007, Fire Testing Technology, London, UK) in accordance with ISO 5660-1. The tangential sections of the wood specimens (100_R_ mm × 10_T_ mm × 100_L_ mm, 3 replicates per group) were irradiated with a heat flux of 50 kW/m^2^ in the vertical direction, and the residues of the wood samples were carefully preserved. 

**Figure 2:**
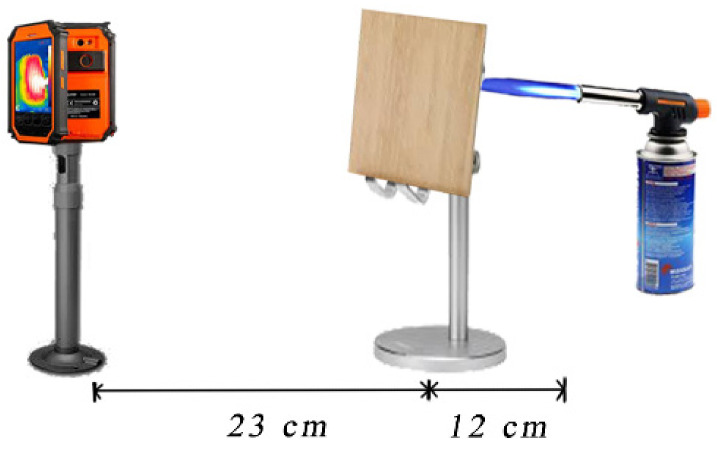
Ultrahigh-temperature stimulation measuring set-up using a butane blowlamp torch.

Moreover, we designed an ignition test simulating the actual fire scene for an intuitive evaluation of the flame retardancy of the wood samples. As shown in Figure 2, a wood panel (100_R_ mm × 10_T_ mm × 100_L_ mm) was fixed vertically between a butane blowlamp and a thermal infrared imager (ST9450, Smart Sensor Intell Instruments, Hong Kong, China). Consequently, the flame of the butane blow torch was always equal in height and strength. The front surface of the panel was directly exposed to the flame. We also captured the pseudocolor thermal imagers of the back surface by using a thermal imager every 1 min during the test. The ignition lasted for 3 min, and Pristine Wood was continuously ignited for 2 min because of its tendency to easily burst into flames and progress to the “out of control” status. The appearances of the front and back surfaces of the wood were ultimately recorded. 

### 2.10. TG-FTIR Analysis 

The gas composition of the wood during thermal degradation was analyzed by TG-FTIR (TG 209F3, NETZSCH, Selb, Germany; iS10 FT-IR spectrometer, Thermo Nicolet Co., Ltd., Madison, WI, USA) in a nitrogen atmosphere, at a heating rate of 10 °C/min from 35 °C to 800 °C. The wood was ground into 200-mesh flour prior to the TG-FTIR detection.

### 2.11. Char Residue Analysis 

The micromorphology of the residue after the CCT was observed by field-emission scanning electron microscope (FE-SEM, SU8010, Hitachi, Tokyo, Japan) with an acceleration voltage of 3–5 kV. Slices measuring approximately 5_R_ mm × 5_T_ mm × 1_L_ mm were cut off from the residues by using a feather microtome blade (3–70, Feather Safety Razor Co., Ltd., Osaka, Japan). The observed surfaces had been sputtered with a thin gold layer before the test.

The chemical composition of the residue was investigated by FTIR (Nicolet iS10, Thermo Nicolet Corporation, Madison, WI, USA) spectroscopy in the 4000–400 cm^−1^ range, with a resolution of 4 cm^−1^ after 64 scans. The wood was ground into 200-mesh flour prior to the FTIR detection. 

### 2.12. Mechanical Property Tests 

The compressive strengths (perpendicular to the grain of the wood in the radial direction, five replicates per group, denoted by σ_1_; parallel to the grain of the wood, eight replicates per group, denoted by σ_2_) of the wood samples measuring 20_R_ mm × 20_T_ mm × 30_L_ mm were determined by universal mechanical testing (MMW50, Naier Testing Machine Co., Ltd., Jinan, China) in accordance with GB/T 1935-2009 and GB/T 1939-2009. The loading rate was 10 mm/min. Hardness test was performed on the cross sections and tangential sections of the wood samples measuring 20_R_ mm × 20_T_ mm × 20_L_ mm by using a shore durometer (TH-210, SDCH Co., Ltd., Beijing, China). The hardness for each group of wood was determined by calculating the average of 20 hardness values for each sample of each group. 

## 3. Results and Discussion

### 3.1. Chemical Structure Analysis

#### 3.1.1. FTIR Analysis

**Figure 3:**
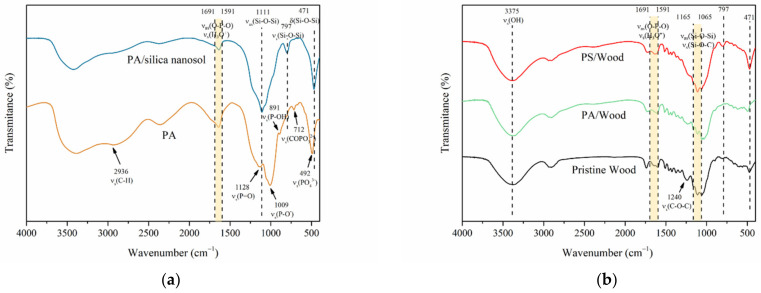
FTIR spectra of (**a**) pure PA, the prepared PA/silica nanosol after heat treatment at 80 °C and (**b**) the wood samples.

Figure 3 displays the FTIR spectra of the pure PA, the prepared PA/silica nanosol with 80 °C heat treatment, and the wood samples. As shown in Figure 3a, the absorption peaks near 2936, 1128, 1009, 891, 712, and 492 cm^−1^, are attributed to the stretching vibrations of C-H, P=O, P-O^−^, P-OH, COPO_3_^2−^, and PO_4_^3−^ structures from the PA molecules, respectively [40,41]. Three characteristic peaks at 471, 797, and 1111 cm^−1^ are assigned to different modes of vibration for Si-O-Si [42,43,44]. From the spectrum of the PA–silica sol, the vibrations of P-O^−^, P-OH, and COPO_3_^2−^ disappeared, indicating the incorporation of the PA molecules into the silica network. 

Figure 3b compares the FTIR spectra of the wood. From the spectrum of Pristine Wood, the peaks near 3375 and 1240 cm^−1^ are assigned to hydroxyl groups (-OH) and ether bonds (C-O-C) in cellulose/hemicellulose, respectively [45]. The 1591–1165 cm^−1^ range represents the fingerprint region of lignin [46]. All characteristic peaks belonging to PA or/and PA–silica sol could be found in the spectra of the correspondingly treated wood. The intensity of either C-O-C or the lignin groups of PA/Wood weakened relative to that of Pristine Wood, revealing hydrolysis reactions of cellulose and hemicellulose of the wood and the decomposition of lignin [31,34]. The presence of Si-O-Si and Si-O-C in PS/Wood indicated that a stable silica network was embedded within the wood scaffold [47,48]. The existence of the silica network in wood may reduce the negative effects of PA on the mechanical properties of wood.

#### 3.1.2. XRD Analysis

**Figure 4:**
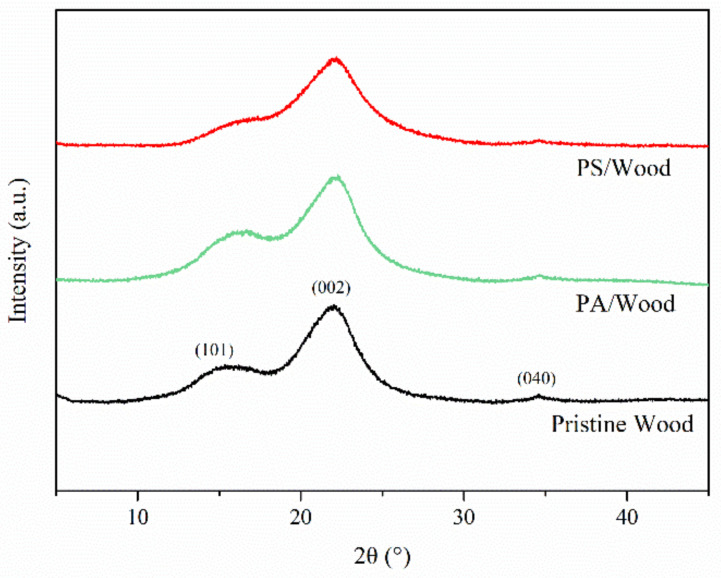
XRD patterns of the wood samples.

Figure 4 shows the XRD patterns of the wood samples. Table 2 lists the calculated crystallinities of the woods. The (101), (002), and (040) planes were observed near 16°, 22°, and 34.5°, respectively, which are ascribed to the cellulose Iβ of wood [49]. However, the intensities of the diffraction peaks of PS/Wood were lower than that of Pristine Wood. This difference may be attributed to the incorporation of silica in the wood matrix, as reported in previous studies [26,27]. The crystallinity of PA/Wood was quite close to that of Pristine Wood (46.41%), revealing a slight change in the crystalline zone of the wood. Meanwhile, PS/Wood exhibited higher crystallinity (50.83%) than that of Pristine Wood, suggesting a more ordered array of cellulose, which rendered the crystal lattices more resistant to shear force during grinding [50,51]. 

### 3.2. Morphology Observation and Element Analysis

**Figure 5:**
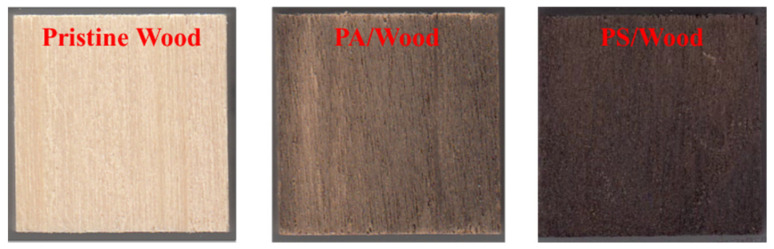
Overall appearances of the wood samples.

The overall appearances of the wood samples are presented in Figure 5. Pristine Wood showed a pale yellow color, whereas PA/Wood and PS/Wood became darker and exhibited brownish tones. These changes can be attributed to the migration and diffusion of extractives on the wood surface [52].

**Figure 6:**
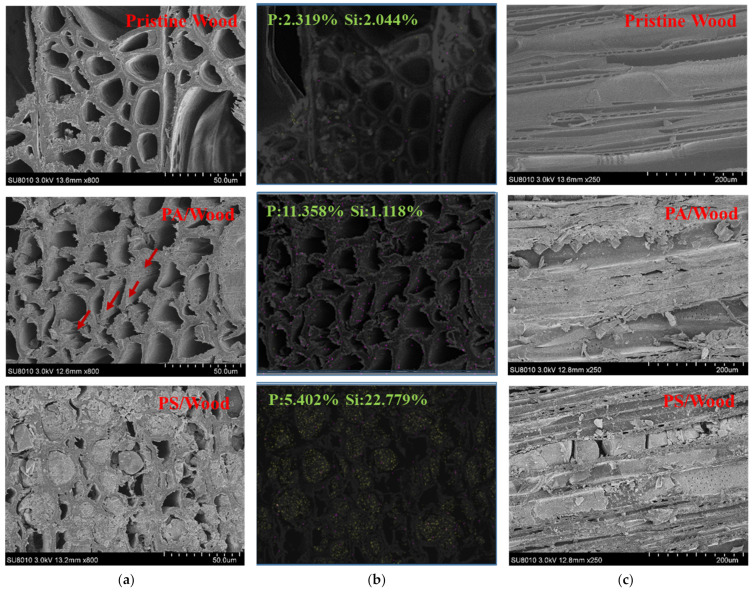
SEM micrographs of the wood samples: (**a**) cross sections (×800 magnification); (**b**) EDS tomographies of the cross sections, purple represents the P element, and yellow represents the Si element; (**c**) tangential sections (×250 magnification).

Figure 6 presents the SEM-EDS micrographs of the wood samples. The cell lumens in Pristine Wood appeared hollow, with only small amounts of P and Si elements detected. The scaffold of PA/Wood was penetrated by PA, but its structure was severely deformed—the cell wall collapsed, and the tissues were split along the seams. These changes resulted from the decomposition of carbohydrates in the wood under hydrothermal conditions in an acidic environment [53]. By contrast, PS/Wood retained an intact wood structure. The pH value of PA–silica sol was similar to that of the PA solution (Table S1) in Supplementary Materials. The stable silica network embedded within the wood scaffold may have protected the carbohydrates in the wood to a certain degree [54,55]. The well-preserved scaffold of PS/Wood may positively affect the mechanical properties of wood relative to PA/Wood [56].

### 3.3. Thermal Degradation Analysis

**Figure 7:**
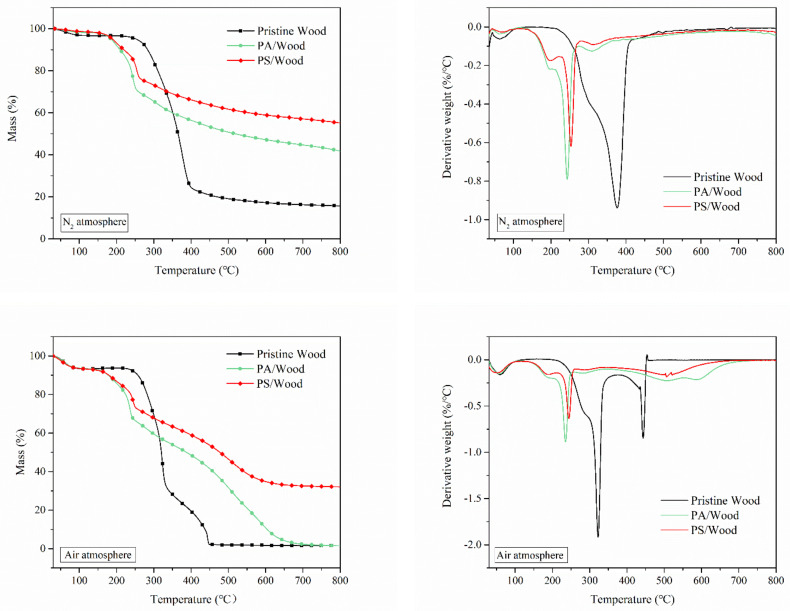
TG and DTG curves of the wood samples in nitrogen and in air.

TG analysis was employed to determine the thermal stability, charring capacity, and decomposition rate of the wood. The TG and derivative thermogravimetric (DTG) curves of the wood samples in nitrogen and air atmosphere are presented in Figure 7. Relevant data associated with the curves are listed in Table 3. 

As shown in Figure 7, the thermal degradation of wood in nitrogen occurred in two steps. The initial mass loss occurred at temperatures below 120 °C because of the evaporation of moisture. As heating follows, a major mass loss occurred within the 240–450 °C range, which resulted from the decomposition of the wood to form flammable gases, volatile liquids, and solid residues [57]. However, in the air atmosphere, the thermal degradation of wood occurred in three steps. The first stage represented the loss of moisture occurring at 30–120 °C. The second stage (150–360 °C) was the main pyrolysis stage and corresponded to the depolymerization of cellulose and hemicellulose of wood into small molecule products. The third stage (360–800 °C) was the further oxidation of the products formed during the second stage in the presence of oxygen.

The initial decomposition of Pristine Wood started at about 283 °C in nitrogen and 257 °C in air, as indicated by *T*_10%_ in Table 3. Compared with Pristine Wood, PS/Wood and PA/Wood showed lower initial decomposition temperatures. This difference was due to the catalytic dehydration and carbonization reactions of wood promoted by phosphorus acid and polyphosphorus acid generated by pyrolysis in the two woods [18]. The presence of preformed char can postpone further pyrolysis of the wood. However, relative to those of PA/Wood, the |*R*_max1_| values of PS/Wood were decreased (21.5% reduction in nitrogen and 21.7% reduction in air), whereas the *T*_max1_ values were increased, suggesting that PS/Wood degradation slowed down at the second pyrolysis stage. Decreases in |*R*_max_| and delays in *T*_max_ were in favor of reduced C-C bond cleavages to allow the formation of more stable char residues during the thermal degradation of wood [58]. At the third stage, further oxidation of PS/Wood was markedly reduced. *T*_max2_ was increased from 443 °C to 522 °C, and |*R*_max2_| was lowered to 0.18%/°C, suggesting that the construction of a PA–silica system can enhance the thermal stability of wood at high temperatures.

Meanwhile, PS/Wood exhibited markedly improved char yields. In nitrogen, the residue achieved a yield of 55.1% at 800 °C, which was twice that of Pristine Wood and 32.1% higher than that of PA/Wood. In air, the residue was 32.1% at 800 °C, whereas those of PA/Wood and Pristine Wood were only 1.5%. These results further demonstrated that the thermal stability of wood was considerably enhanced with PS treatment. The high thermal stability of PS/Wood may result from the synergistic FR effect of P and Si [34]. Whereas P in the degradation products promoted char formation, Si contributed to thermal shielding. 

### 3.4. Combustion Property

#### 3.4.1. Flammability

**Figure 8:**
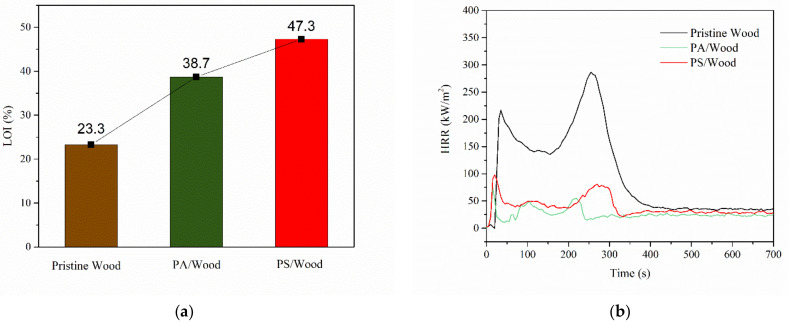
(**a**) LOI values and (**b**) HRR profiles of the wood samples.

The flammability of the prepared wood was assessed based on LOI and CCT. As shown in Figure 8a, the LOI of PS/Wood was 47.3%, and those of Pristine Wood and PA/Wood were 23.3% and 38.7%, respectively. PS/Wood exhibited the higher LOI, suggesting a more improved flame retardancy of the wood in a small-scale flame scenario compared with PA/Wood.

The heat release rate (HRR) profiles of the wood samples obtained from CCT are presented in Figure 8b, and detailed combustion data are listed in Table 4. As shown in Figure 8b, the HRR curve of Pristine Wood showed a typical two-peak-profile characteristic for wood as a thermally thick-charring material [38]. The first HRR peak (PHRR_1_) appeared immediately after ignition, owing to the pyrolysis and combustion of the surface wood. A char layer was subsequently formed. The second peak (PHRR_2_) appeared, which was attributed to char cracking and pyrolysis of the inner wood. However, middle peaks were present in PA/Wood and PS/Wood, which may be attributed to small cracks on the char layer and led to the pyrolysis of the inner wood. Pristine Wood showed PHRR_1_ equal to 201.8 kW/m^2^ at 38 s and PHRR_2_ equal to 250.8 kW/m^2^ at 280 s. Compared with those of Pristine Wood, the PHRR_1_ and PHRR_2_ values of PA/Wood and PS/Wood were markedly reduced—those of PA/Wood were decreased by 63.8% and 81.8%, respectively, and those of PS/Wood were decreased by 55.8% and 69.5%, respectively. The PHRRs and total heat release (THR) of PS/Wood were slightly higher than those of PA/Wood. This small difference may be ascribed to the presence of more organic residues in PS/Wood than in PA/Wood [59]. Notably, the PHRR_2_ occurrence time of PS/Wood was postponed, whereas that of PA/Wood occurred earlier, which was attributed to the presence of noncombustible silica and the improved thermal stability of the formed char layer. Therefore, the mass loss of PS/Wood was 17.8% lower than that of PA/Wood and nearly 37.4% lower than that of Pristine Wood. In the designed ignition test, PS/Wood also exhibited distinct flame retardancy. No cracks were found on the back surface at the end of the burning process, whereas certain degrees of damage to the back surfaces of Pristine Wood and PA/Wood were found (Figure S2) in Supplementary Materials.

#### 3.4.2. Fire Toxicity

**Figure 9:**
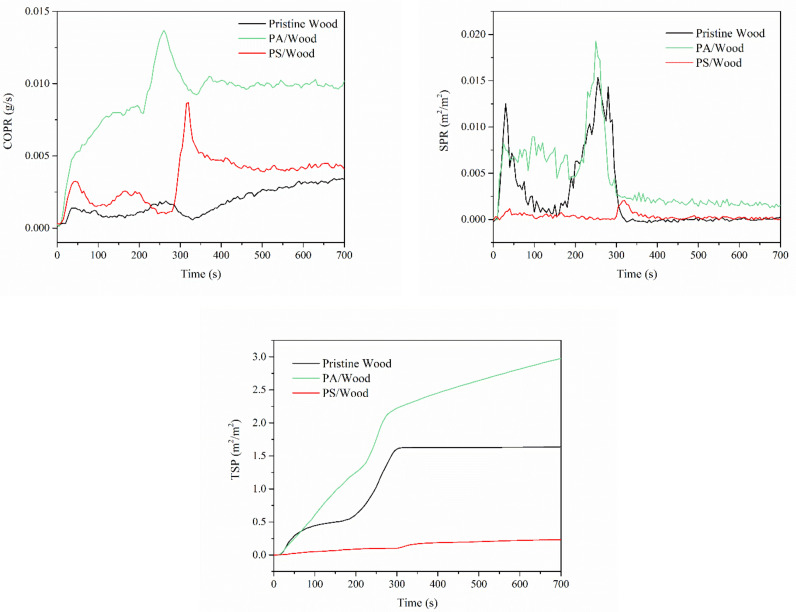
COPR, SPR, and TSP profiles of the wood samples.

As an asphyxiant, CO prevents hemoglobin from binding with oxygen, leading to unconsciousness and even death [60]. Smoke particulates present a respiratory hazard and impair the ability to escape by visible obscuration. Therefore, fire toxicity needs to be estimated in assessing potential fire risks related to inflammable materials. Figure 9 presents the profiles of the CO production rate (COPR), smoke production rate (SPR), and total smoke production (TSP) of the wood samples. Table 5 lists other CO and smoke parameters determined by CCT. 

As shown in Figure 9, CO and smoke were produced after the ignition of wood, leading to COPR and SPR peaks. When the surface of the wood is ignited, oxygen penetrated the wood through cracks, hence the appearances of other COPR and SPR peaks [54]. Pristine Wood had considerably low COPR values but rather high SPR values during combustion, with COY/CO2Y of 0.02 and TSP of 1.6 m^2^/m^2^. Compared with those of Pristine Wood, the COPR and CO yield of PA/Wood markedly increased owing to the incomplete combustion of the treated wood samples after the formation of the protective char layer [61]. Meanwhile, the slope of the TSP curve of PA/Wood was higher than that of Pristine Wood, indicating that the PA-treated wood produced more smoke than that of Pristine Wood. The slope of the COPR curve of PS/Wood was considerably lower than that of PA/Wood, and the SPR and TSP values were markedly reduced relative to those of Pristine Wood and PA/Wood. Smoke release can be impeded by a highly thermostable carbonized layer with silica [62].

In summary, these results demonstrated that the construction of the PA–silica system in wood conferred excellent flame retardancy and smoke-suppression properties on wood. 

### 3.5. Flame Retardancy and Smoke-Suppression Mechanism 

#### 3.5.1. TG-FTIR Analysis

**Figure 10:**
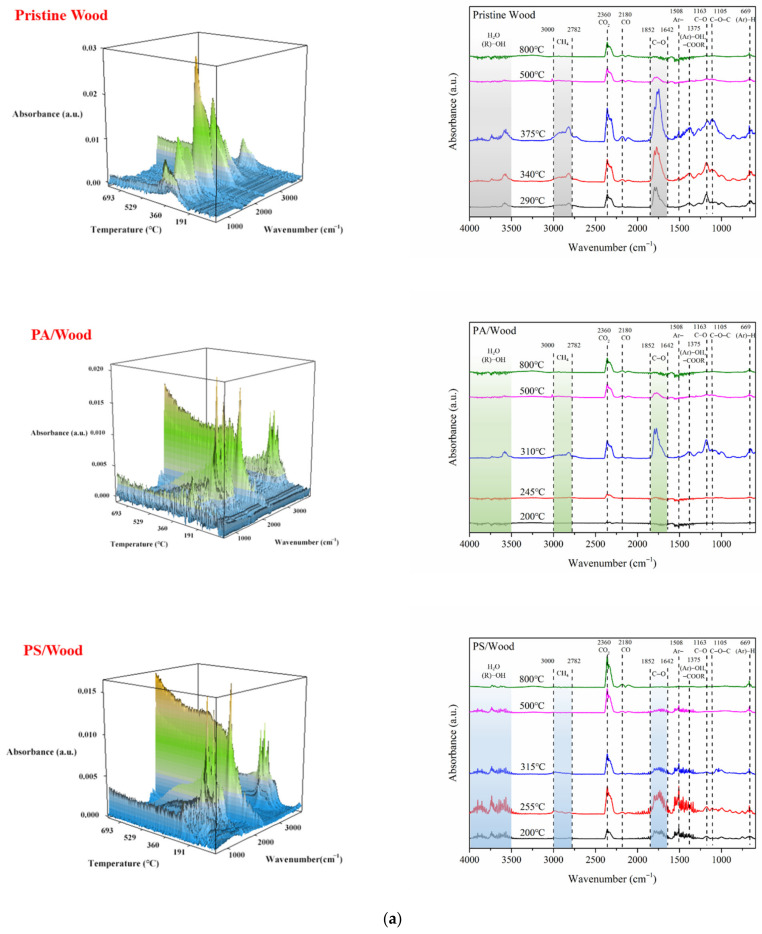

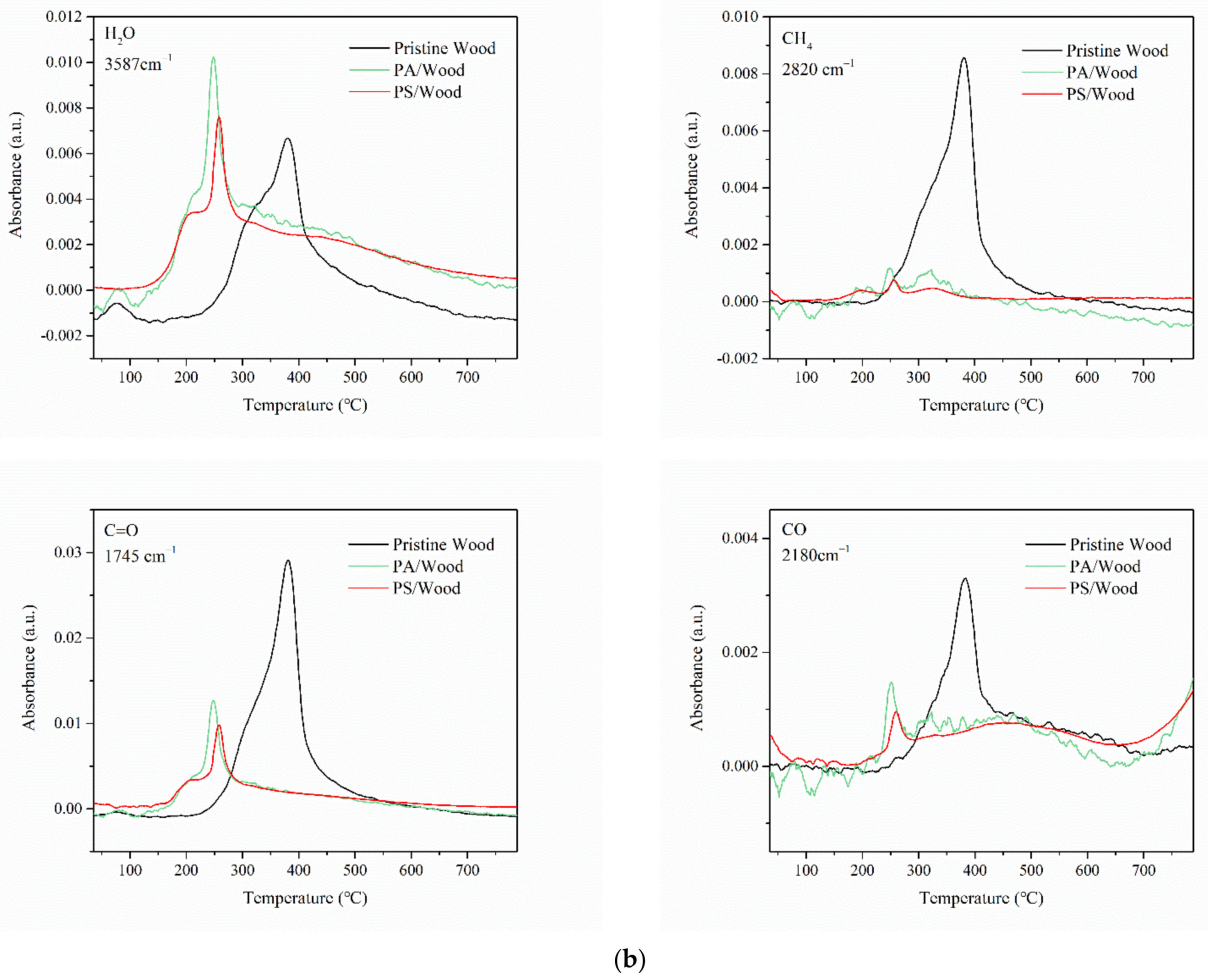
FTIR spectra of (**a**) the evolved gases, and (**b**) contents of H_2_O, CH_4_, C=O and CO produced during the whole pyrolysis process of the wood samples.

The gas composition of the wood during thermal degradation was analyzed by TG-FTIR. Figure 10a shows the gas FTIR spectra of the wood at increased temperatures. The temperature points in each plane figure were chosen based on the corresponding TG curves of the wood (in nitrogen) presented in Figure 7. 

The absorption peaks detected at 3587, 2820, 2360, 2180, 1745, 1508, 1163, 1105, 984, and 669 cm^−1^ are ascribed to the vibrations of H_2_O, CH_4_, CO_2_, CO, C=O, Ar-, C-O, C-O-C, and C-H (in aromatic hydrocarbon) structures, respectively [63,64,65,66]. The peaks at 1375 cm^−1^ are assigned to the stretching vibration of phenolic hydroxyl groups, which overlap with the vibration of ester groups. Under heating conditions, wood releases free water and bound water. As a further increase in temperature, dehydration and carbonization reactions occur in wood, along with the production of moisture, flammable volatiles, and smoke. From 250 °C to 530 °C, methane was released from cleavages of carbon chains either by C-C and β-β scission or aryl-ether cleavage [67], and carbonyl was derived from the decarbonylation reaction of hemicellulose [68]. Simultaneously, aromatic hydrocarbon was generated from the decomposition of lignin and aromatization of lignocellulose. C-O, C-O-C, and C-H groups were derived from dehydration, decarboxylation, or decarbonylation reactions. CO was derived from the restructuring and isomerization reactions of wood components, and CO_2_ was derived from the decarboxylation reaction of xylose and complete combustion of carbonaceous matter.

Figure 10b presents the contents of H_2_O, CH_4_, C=O, and CO during the pyrolysis of the wood samples. H_2_O can absorb heat by evaporation and thus can reduce the temperature of the whole system. Pristine Wood started to produce a large amount of water when the temperature reached 264 °C. Compared with Pristine Wood, PS/Wood and PA/Wood produced much water earlier during pyrolysis as a result of the dehydration reaction of cellulosic substances catalyzed by phosphorus elements from PA [19]. CH_4_ and C=O are flammable gases that cause fire to spread when mixed with air. As shown below, CH_4_, C=O, or CO produced by Pristine Wood had an extremely high content. The amounts of gas containing CH_4_, CO, or C=O groups produced by PS/Wood and PA/Wood were markedly reduced relative to those produced by Pristine Wood, suggesting that the carbon chains had less cleavages to form flammable gases (small molecules) [18]. These results indicated that the construction of the PA–silica system could reduce the flammable and toxic gases released from wood during pyrolysis.

#### 3.5.2. Char Analysis

**Figure 11:**
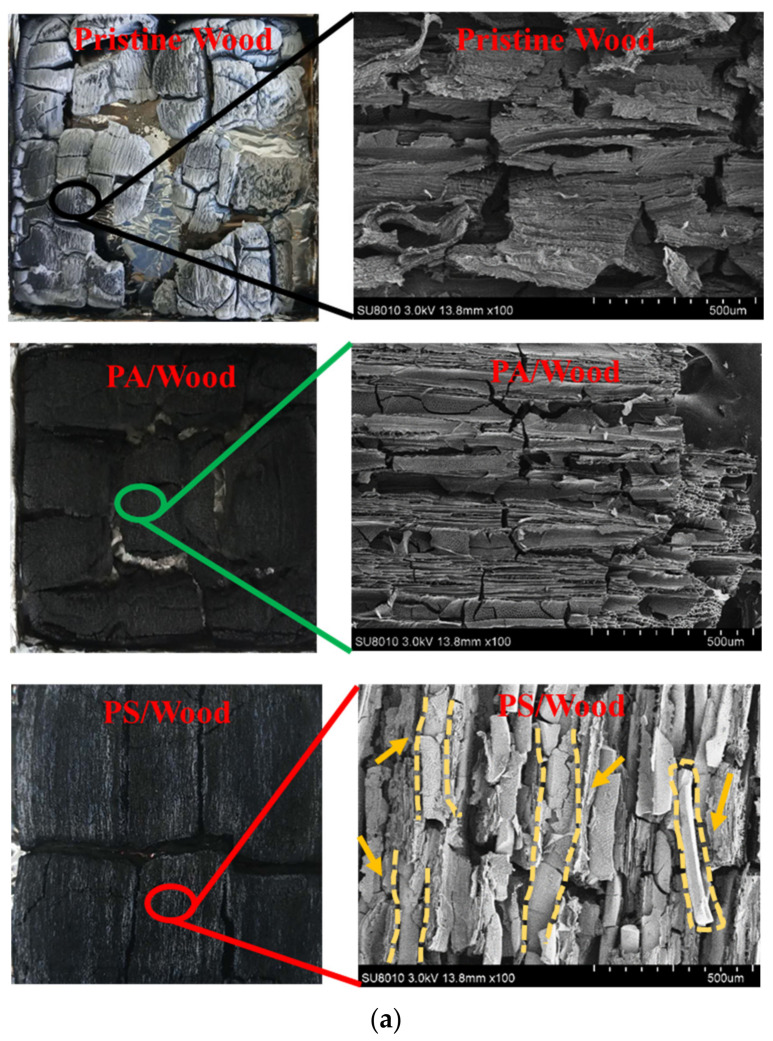

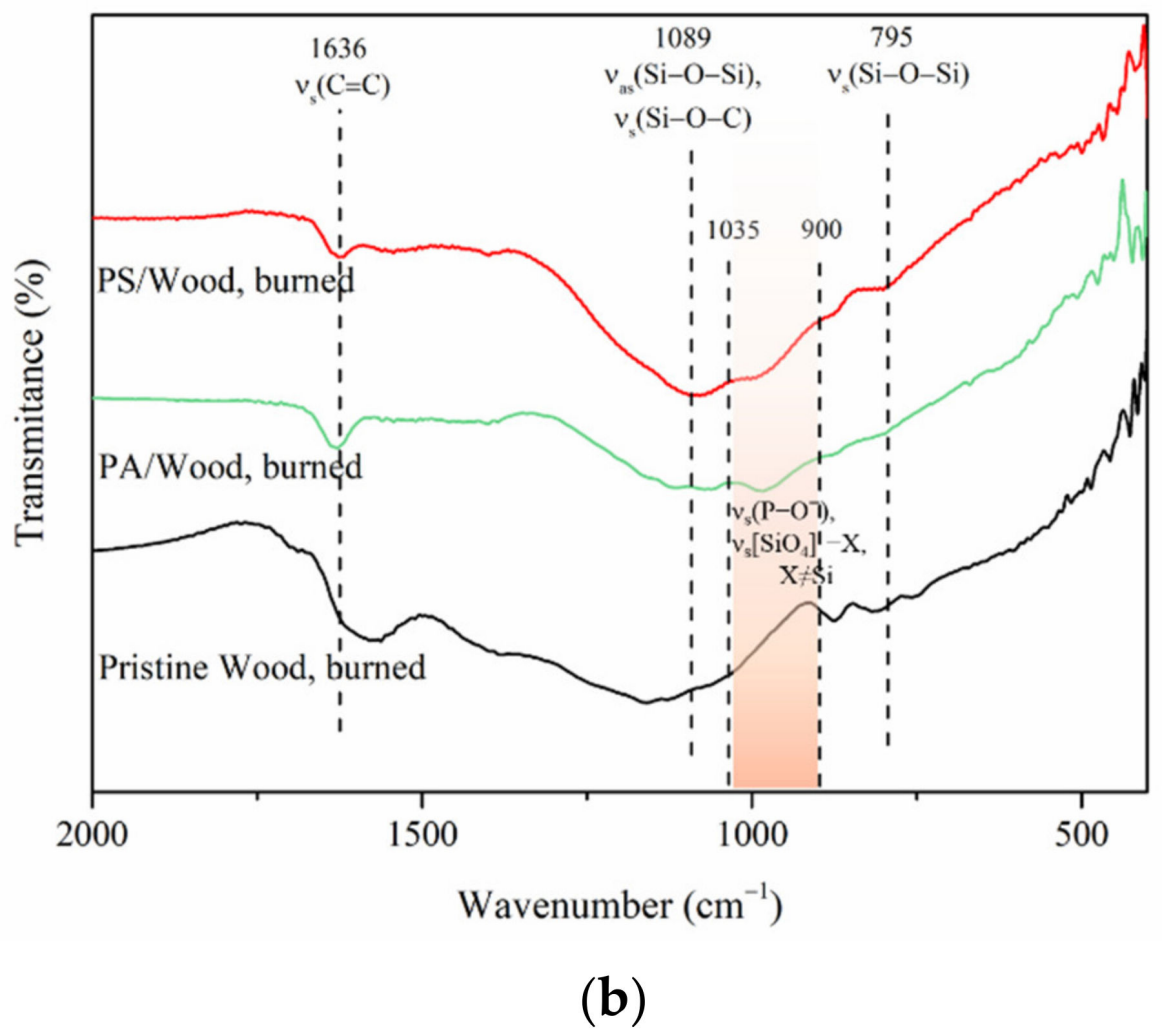
(**a**) Digital photos, SEM micrographs (×100 magnification) and (**b**) FTIR spectra of the char residues after CCT.

SEM and FTIR measurements were conducted to investigate the characteristics of the char residues after CCT. As shown in Figure 11a, a thick layer of grey–white ash was observed in the residue of Pristine Wood. Char was largely disintegrated, revealing the violent combustion of the wood. The char residue of PA/Wood was incompact and crumbly, showing numerous extensive cracks on its surface. This condition resulted from the low thermal stability of P-catalyzed char at high temperatures [20]. In Figure 6, the deformed structure of PA/Wood before combustion may be the other reason for the cracks. Consequently, large amounts of CO and smoke escaped from the cracks, thereby increasing the COPR, SPR, and TSP, as determined by CCT. However, the char residue of PS/Wood was more intact and coherent. Numerous white, ribbon–shaped solids were covered on the microcracks of the char. These substances could block the cracks generated by wood decomposition, more efficiently inhibiting the release of heat and smoke [69]. 

Figure 11b compares the FTIR spectra of the wood residues after CCT. The peaks at 1636 cm^−1^ are assigned to C=C structures [70]. More C=C structures were retained in the residue of PS/Wood and PA/Wood relative to that of Pristine Wood, revealing the reduced decomposition of the wood components [71]. From the FTIR spectrum of the PS/Wood residue, the peak at 900–1035 cm^−1^ is ascribed to SiO_4_ stretches perturbed by the presence of a neighboring atom other than Si, which is overlapped by the peak of P-O^−^ structures [32]. These two peaks were covered by the broad peak of Si-O-C and Si-O-Si structures, both indicating the formation of crosslinked char residue during the combustion of PS/Wood [21,54]. 

#### 3.5.3. Mechanism Analysis

Given the aforementioned results, we concluded that the constructed hybrid system conferred highly efficient flame retardancy and smoke-suppression properties on wood. The potential mechanism may be explained as follows. In a flame scenario, phosphorus elements from PA catalyzed the dehydration of cellulosic substances and promoted char formation in the initial stages of pyrolysis [20]. The protective char residue on the wood surface could prevent the underlying wood substrate from direct exposure to the flame [72]. The incorporated silica then enhanced the thermal stability of the char residue in two ways: (i) It densely covered the carbonaceous char, rendering the whole residue more intact and coherent, and protected the wood substrate from any contact with heat and oxygen [73]; and (ii) at high temperatures (>450 °C), it could form crosslinked structures with P [32,74], which were more thermally stable and difficult to decompose [75]. In this case, the total residues (carbonaceous char + silica/phosphosilica) of PS/Wood acted as physical barriers to heat and mass transfer, which not only enhanced protection but also sustained the whole residue under a fully developed fire. Therefore, heat release and smoke production were efficiently inhibited.

### 3.6. Mechanical Property 

**Figure 12:**
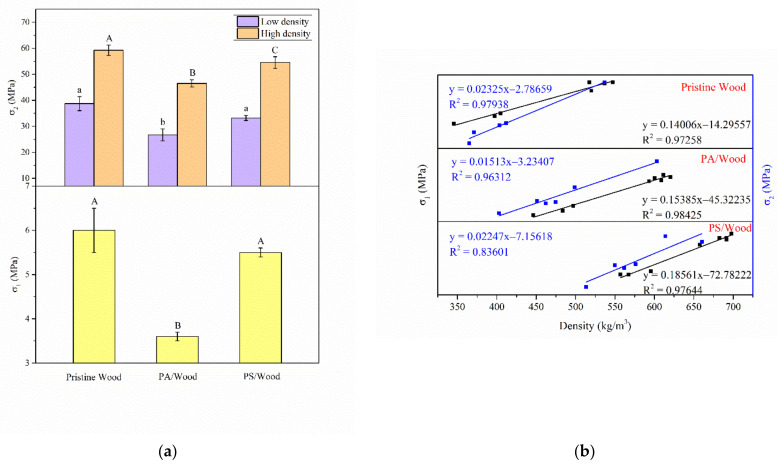

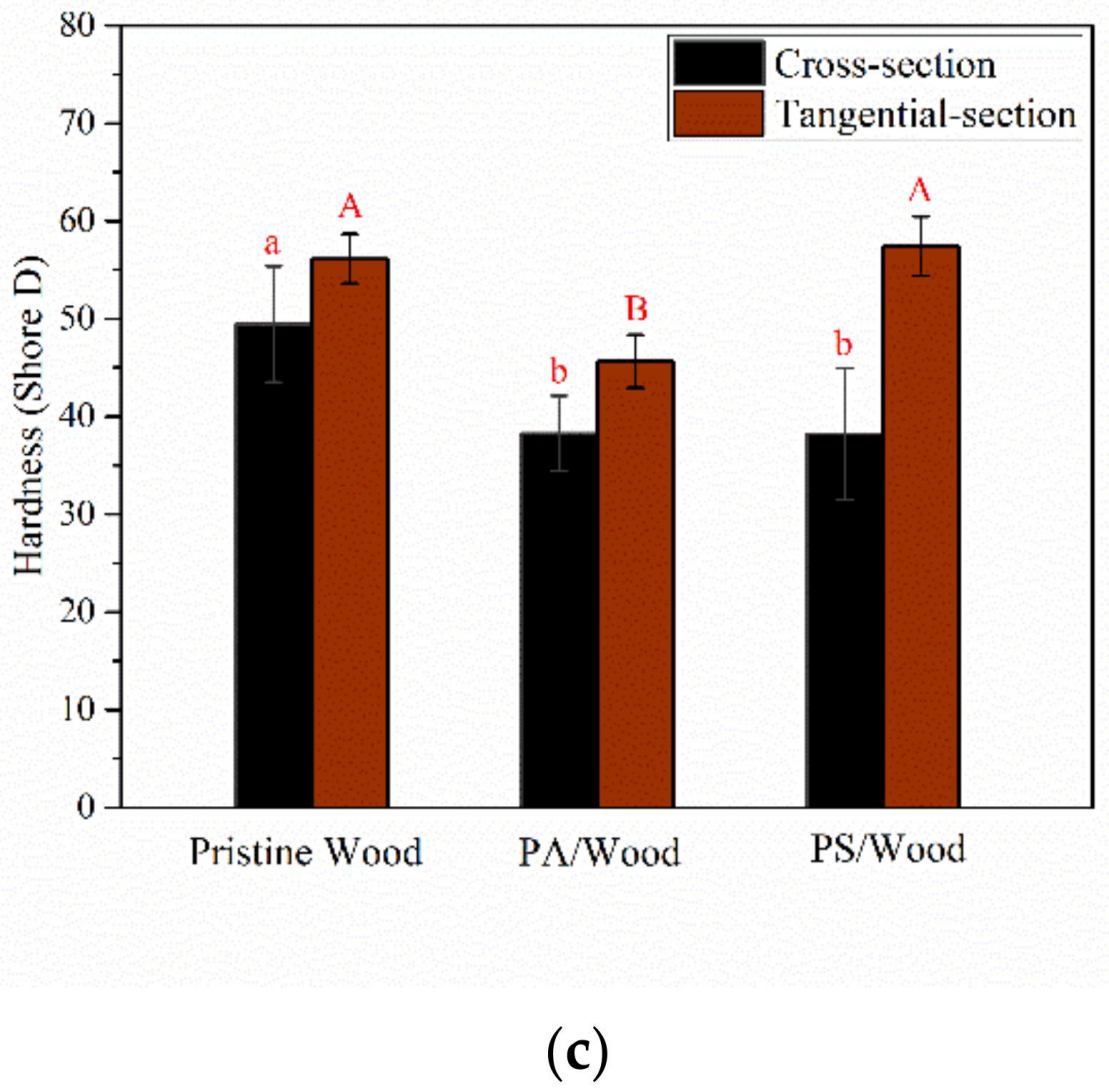
(**a**) Compressive strength (σ_1_: perpendicular to the grain in the radial direction; σ_2_: parallel to the grain) of the wood samples; (**b**) dependencies of σ_2_ on the density of each treated wood sample for different experimental groups; (**c**) shore hardness of the wood samples. Bars with different letters indicate a significant difference (*p* < 0.05) in accordance with Duncan’s multiple range test.

In this section, the effects of the PA–silica system on the mechanical properties of wood, such as compressive strengths and surface hardness, are evaluated. The results are shown in Figure 12. The compressive strength perpendicular to the wood grain in the radial direction and parallel to the wood grain were denoted by σ_1_ and σ_2_, respectively. To improve the comparison, the samples used in the σ_2_ test were divided into two pretreatment groups: low-density group and high-density group. In the low-density group, the densities of the wood before treatment were in the 344–412 kg/m^3^ range; in the high-density group, the densities of the wood were in the 504–539 kg/m^3^ range. 

The σ_1_ value of Pristine Wood was 6.0 MPa, and the σ_2_ values were 38.7 MPa and 59.2 MPa for the low-density and high-density groups, respectively. A higher density of wood typically indicates better mechanical properties [76]. In the current study, the densities of the wood improved after FR treatment. Nonetheless, PA/Wood showed inferior mechanical properties to those of Pristine Wood—the σ_1_ and σ_2_ values were reduced by 40.7% and 21.4–40.0%, respectively, and this reduction was even more notable for the low-density group (Figure 12a). Moreover, the cross-section and tangential-section hardness of Pristine Wood were 49.5 and 56.1 HD, respectively. However, the surface hardness of PA/Wood was decreased relative to that of Pristine Wood, reflecting corresponding reduction of 29.3% in the cross section and 18.7% in the tangential section (Figure 12c). These results can be ascribed to the deterioration of the wood components, which led to the deformation of the structure of PA/Wood. The σ_1_ and σ_2_ values of PS/Wood increased by 52.7% and 17.2–24.3%, respectively, and the tangential-section hardness was increased by 11.1%, relative to that of PA/Wood. These findings indicated a more beneficial effect of the constructed hybrid system on the mechanical properties of wood than that of the pure PA system.

The compressive strength as a function of wood density in different experimental groups is presented in Figure 12b. Overall, the compressive strength increased linearly with the density of the wood sample. The correlation coefficients (*R^2^*) ranged from 0.84 to 0.99. With an increase in density, Pristine Wood slowly increased in σ_1_ and σ_2_. The gaining rate in the σ_1_ value of PA/Wood slightly improved relative to that of Pristine Wood, but the gaining rate of σ_2_ was largely decreased. By contrast, with an increase in density, PS/Wood most rapidly increased in σ_1_ and σ_2_, also indicating the superiority of the mechanical properties of the constructed hybrid system on wood than those of the pure PA system. 

The improvement in the resistance of PS/Wood to deformation and collapse may be ascribed to the incorporation of the silica network, which embedded itself within the wood scaffold. Thus, the density and hardness of the wood were increased relative to that of PA/Wood.

## 4. Conclusions

In this study, a PA–silica system was successfully constructed in wood by a vacuum-pressure impregnation process. FTIR and SEM-EDS results indicated that PA–silica sol was incorporated into the wood, and a stable network was embedded within the wood scaffold. Thus, the structure of PS/Wood was well retained. TG analysis showed that phosphorus elements from PA could catalyze dehydration of cellulosic substances in wood, as well as promote char formation in the initial stages of pyrolysis; simultaneously, the construction of the hybrid system greatly enhanced the thermal stability of the wood. Combustion property tests revealed that the system exhibited highly efficient flame retardancy and smoke-suppression properties. The LOI of PS/Wood reached 47.3%. The PHRRs of PS/Wood were significantly decreased by 55.6 and 69.5% relative to those of Pristine Wood. Meanwhile, the times to PHRRs, which were 20 and 282 s, were greatly delayed relative to those of PA/Wood (15 and 222 s). The production of CO and smoke was much less than that of PA/Wood. The COY/CO_2_Y and TSP values were decreased by 56.5 and 90%, respectively, compared with those of PA/Wood. The superior flame retardancy and smoke suppression of the hybrid system were mainly attributed to the generation of more intact and compact char residue rich in crosslinked structures. Moreover, the constructed hybrid system did not significantly alter the mechanical properties of wood, such as compressive strength and hardness. Owing to environmentally friendly characteristics, this constructed PA–silica system in wood developed using a simple top-down process shows great potential for application in fire-safety fields.

The particle size of the sol is highly related to its penetration in wood. However, the average particle size of the hybrid sol was 33.8 nm, and the PA/silica particles were mainly located in the cell lumens of wood. The lack of penetration of sol into cell wall would limit the formation of bonds with it, thus limiting an improvement of the thermal properties of wood. Further study is expected concerning treating wood with PA/silica nanosol with small particle size less than 2 nm. 

## Figures and Tables

**Table 1:** Impregnations and curing treatments (gelation and drying) for different experimental groups.

Sample	Impregnation					Curing			
	Impregnates	Vaccum Phase		Pressurized Phase		Gelation		Drying	
		Pressure (MPa)	Time (min)	Pressure (MPa)	Time (h)	Temperature (°C)	Time (d)	Temperature (°C)	Time (d)
Pristine Wood	Deionized water	−0.09	30	0.50	2	25	2	80	3
PA/Wood	PA solution	−0.09	30	0.50	2	25	2	80	3
PS/Wood	PA/silica sol	−0.09	30	0.50	2	25	2	80	3

**Table 2:** Relative crystallinities of the wood samples.

Sample	Relative Crystallinity (%)
Pristine Wood	46.41
PA/Wood	45.05
PS/Wood	50.83

**Table 3:** Relevant data from the TG and DTG curves in nitrogen and air.

**Atmosphere: N_2_**						
**Sample**	***T*_10%_ (°C)**		***T*_max1_ (°C)**		**|*R*_max1_| (%/°C)**	**Residue@800 °C** **(%)**
Pristine Wood	283		376		0.94	15.7
PA/Wood	209		244		0.79	41.7
PS/Wood	219		254		0.62	55.1
**Atmosphere: air**						
**Sample**	***T*_10%_ (°C)**	***T*_max1_ (°C)**	**|*R*_max1_| (%/°C)**	***T*_max2_ (℃)**	**|*R*_max2_| (%/°C)**	**Residue@800 °C** **(%)**
Pristine Wood	257	323	1.91	443	0.85	1.5
PA/Wood	178	235	0.89	507	0.23	1.5
PS/Wood	180	244	0.63	522	0.18	32.1

*T*_10%_: temperature at 10% mass loss; *T*_max_: temperature at the maximum mass-loss rate; *R*_max_: maximum decomposition rate.

**Table 4:** Combustion data for the wood samples during CCT.

Sample	PHRR_1_ (kW/m^2^)	Time to PHRR_1_ (s)	PHRR_2_ (kW/m^2^)	Time to PHRR_2_ (s)	THR (MJ/m^2^)	MEHC (MJ/kg)	Mass Loss (%)
Pristine Wood	201.8 ± 10.6	38 ± 8	258.0 ± 67.9	280 ± 25	68.2 ± 3.3	14.2 ± 0.4	86.15 ± 0.55
PA/Wood	73.0 ± 4.5	15 ± 0	47.0 ± 5.7	222 ± 6	24.6 ± 1.1	5.6 ± 0.3	65.58 ± 1.07
PS/Wood	89.6 ± 6.4	20 ± 0	78.6 ± 13.9	282 ± 24	34.2 ± 5.8	8.0 ± 1.2	53.91 ± 0.72

PHRR: peak of heat release rate; THR: total heat release; MEHC: mean effective heat combustion.

**Table 5:** CO and smoke parameters of the wood samples during CCT.

Sample	FR Loading (kg/m^3^)	Time to PCOPR (s)	COY/CO_2_Y	MSEA (m^2^/kg)	Time to PSPR_1_ (s)	Time to PSPR_2_ (s)	TSP (m^2^/m^2^)
Pristine Wood	-	283 ± 19	0.02 ± 0.00	36.2 ± 12.7	32 ± 6	277 ± 21	1.6 ± 0.6
PA/Wood	76.8 ± 0.7	263 ± 8	0.23 ± 0.02	77.5 ± 7.7	28 ± 2	250 ± 8	3.0 ± 0.3
PS/Wood	181.4 ± 5.0	342 ± 24	0.10 ± 0.02	7.5 ± 0.5	40 ± 0	320 ± 16	0.3 ± 0.0

PCOPR: peak of CO production rate; COY/CO_2_Y: ratio of CO yield to CO_2_ yield; MSEA: mean specific extinction area; PSPR: peak of smoke production rate; TSP: total smoke production.

## Data Availability

The data presented in this study are available upon request from the corresponding author.

## Supplementary Materials

The following are available online at https://www.mdpi.com/article/10.3390/ma14154164/s1, Table S1: Formulations for wood impregnation and physical properties of the correspondingly treated wood, Figure S1: TG and DTG curves of pure PA and the prepared PA/silica nanosol after heat treatment at 80 °C in air, Figure S2: Infrared images of the back surfaces of the woods after continued ignition at 1, 2, and 3 min and self-extinction at 5 min; front and back surfaces of the wood samples appearing at the end of the ignition test.
