# Supplementary material for: Construction of a Phytic Acid–Silica System in Wood for Highly Efficient Flame Retardancy and Smoke Suppression
Source: Materials (Basel). 2021 Jul 27;14(15):4164. doi: 10.3390/ma14154164 (PMC8347795; doi:10.3390/ma14154164)
Supplement: Supplementary file 1 [file materials-14-04164-s001.zip › materials-1267660-supplementary.pdf]

materials MDPI

Supplementary Materials

Construction of a Phytic Acid–Silica System in Wood for Highly Efficient Flame Retardancy and Smoke Suppression

Zhuoran Chen, Shaodi Zhang, Mengyi Ding, Mingzhi Wang * and Xing Xu

Table S1. Formulations for wood impregnation and physical properties of the correspondingly treated wood.

Scheme	Impregnation solution				WPG (%)	BC (%)	Oven-dried density after treatment (*10^{-3} kg/m^3)	Thermal conductivity [W/(m·K)]
	Mass fraction (%)		Physicochemical properties					
	PA	SiO_2	pH	Average particle size (nm)				
Pristine Wood	-	-	-	-	0.00±0.00	0.00±0.00	0.42±0.01	0.1235±0.0010
PA/Wood	10	0	0.94	< 1	16.80±0.24	7.21±0.61	0.48±0.00	0.1306±0.0026
PS/Wood	10	15	0.95	33.8	24.99±0.94	8.22±0.78	0.51±0.00	0.1313±0.0034

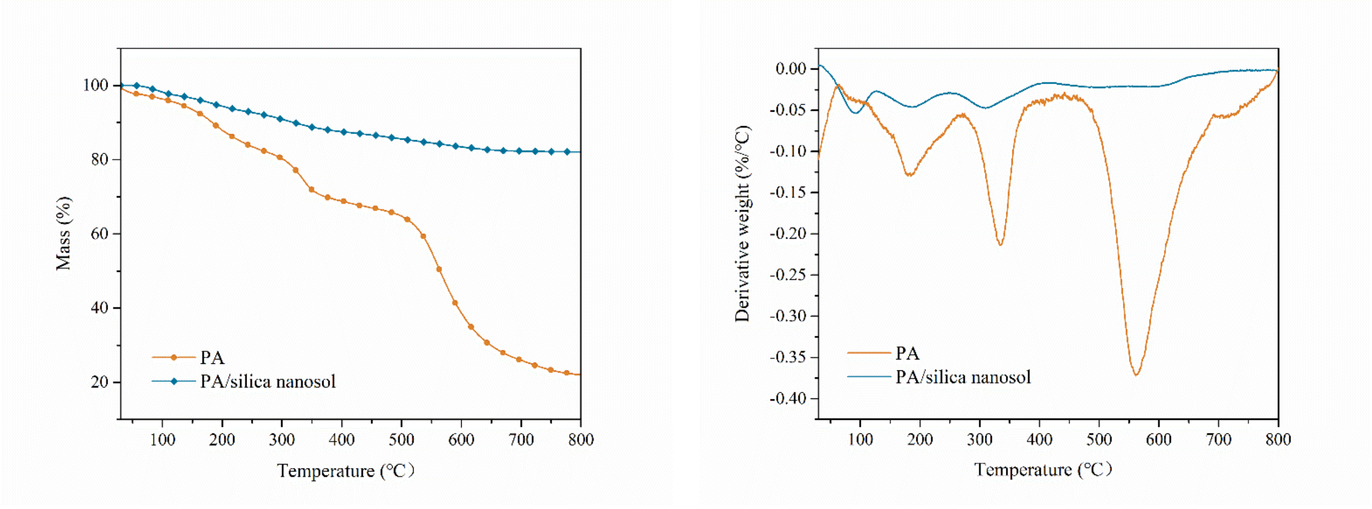

Figure S1. TG and DTG curves of pure PA and the prepared PA/silica nanosol after heat treatment at 80°C in air.

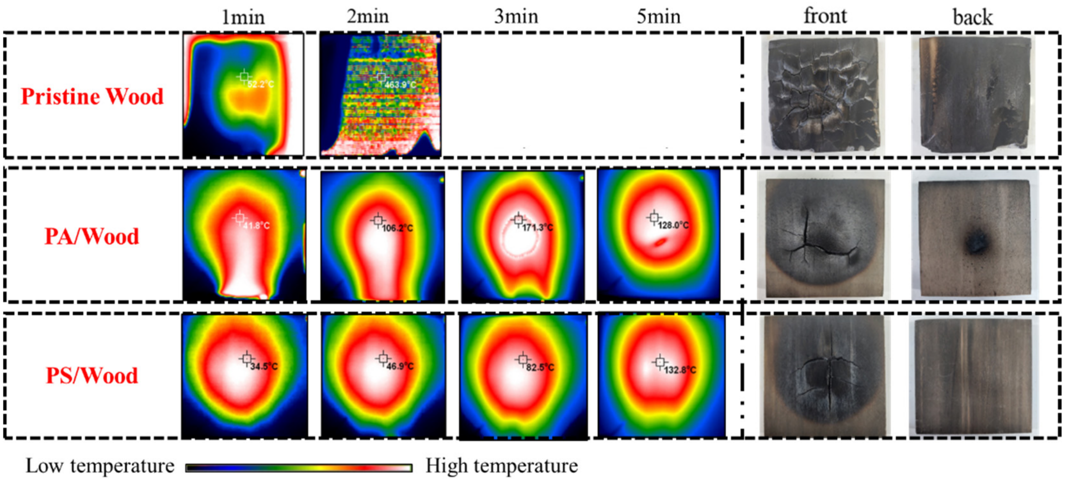

Figure S2. Infrared images of the back surfaces of the woods after continued ignition at 1, 2, and 3 min and self-extinction at 5 min; front and back surfaces of the wood samples appearing at the end of the ignition test.
